# Supplementary material for: Mechanisms governing the pioneering and redistribution capabilities of the non-classical pioneer PU.1
Source: Nat Commun. 2020 Jan 21;11:402. doi: 10.1038/s41467-019-13960-2 (PMC6972792; doi:10.1038/s41467-019-13960-2)
Supplement: Supplementary file 7 — Source data [file 41467_2019_13960_MOESM7_ESM.zip › Source_Data/FigureS2/FigureS2B_MotifScanOutput/KmeansCluster1/homerResults/motif39.similar.html]

motif39

## Information for motif39

C
T
G
A
A
G
C
T
C
T
A
G
C
T
G
A
A
G
C
T
C
G
A
T
A
C
T
G
C
A
G
T
C
G
T
A
C
G
T
A
A
C
T
G
C
G
A
T
  
Reverse Opposite:  

C
G
T
A
G
T
A
C
A
C
G
T
A
C
G
T
G
C
T
A
A
G
T
C
C
G
T
A
T
C
G
A
G
A
C
T
G
A
T
C
C
T
G
A
G
A
C
T
  

|  |  |
| --- | --- |
| p-value: | 1e-18 |
| log p-value: | -4.179e+01 |
| Information Content per bp: | 1.889 |
| Number of Target Sequences with motif | 49.0 |
| Percentage of Target Sequences with motif | 0.96% |
| Number of Background Sequences with motif | 59.8 |
| Percentage of Background Sequences with motif | 0.15% |
| Average Position of motif in Targets | 88.6 +/- 70.6bp |
| Average Position of motif in Background | 92.0 +/- 72.9bp |
| Strand Bias (log2 ratio + to - strand density) | -0.5 |
| Multiplicity (# of sites on avg that occur together) | 1.00 |
| Motif File: | file (matrix) reverse opposite |

### Similar de novo motifs found

|  |  |  |  |  |  |  |  |
| --- | --- | --- | --- | --- | --- | --- | --- |
| Rank | Match Score | Redundant Motif | P-value | log P-value | % of Targets | % of Background | Motif file |
| 1 | 0.928 | C T G A A C G T C T A G C G T A G A C T C G A T A T C G A C G T C T G A T G C A T A C G G A C T A G C T | 1e-17 | -41.299408 | 1.49% | 0.38% | motif file (matrix) |
| 2 | 0.790 | C T G A A C G T A C G T A C T G G C A T C G T A C G T A T C A G C G A T A C G T A G C T | 1e-15 | -35.585355 | 1.28% | 0.33% | motif file (matrix) |
